# Correcting Hardening Artifacts of Aero-Engine Blades with an Iterative Linear Fitting Technique Framework

**DOI:** 10.3390/s24062001

**Published:** 2024-03-21

**Authors:** Yenan Gao, Jian Fu, Xiaolong Chen

**Affiliations:** 1 School of Mechanical Engineering and Automation, Beihang University, Beijing 100191, China; peregrine@buaa.edu.cn (X.C.); 2 Jiangxi Research Institute, Beihang University, Nanchang 330000, China; 3 Ningbo Institute of Technology, Beihang University, Ningbo 315800, China

**Keywords:** aero engines, iterative linear fitting, hardening correction, industrial computed tomography, image artifact

## Abstract

Aero engines are the key power source for aerospace vehicles. Cermet turbine blades are the guarantee for the new-generation fighters to improve aero-engine overall performance. X-ray non-destructive reconstruction can obtain the internal structure and morphology of cermet turbine blades. However, the beam hardening effect causes artifacts in objects and affects the reconstruction quality, which is an issue that needs to be solved urgently. This study proposes a hardening-correction framework for industrial computed tomography (ICT) images based on iterative linear fitting. First, an iterative binarization was performed to improve the penetration length accuracy of the forward projection. Then, the proposed linear fitting technology combined with the Hermite function model is derived and analyzed to obtain suitable parameters of blade data. Finally, the fitting curves of the blade data, using the proposed method and the traditional polynomial fitting method, were analyzed and compared and were used to correct the engine turbine blade projection data to reconstruct different groups of tomographic images. Different groups of tomographic images were analyzed using three quantitative image quality evaluation indicators. The results show that the root-mean-square error (RMSE) of the tomographic image obtained by the proposed framework is 0.0133, which is lower than that of the compared method. The peak signal-to-noise ratio (PSNR) is 37.7050 dB and the feature structural similarity (FSIM) is 0.9881, which are both higher than that of the compared method. The proposed method improves the hardening-artifact-correction capability and can obtain higher-quality images, which provides new ideas for the development of imaging and detection of new-generation aero-engine turbine blades.

## 1. Introduction

Aero engines are heat engines, which indicates that their thrust comes from the energy generated by the heated expansion of air. If the temperature in front of the engine turbine is increased by 100 °C, the engine thrust can be increased by about 20%. Research has found that cermet turbine blades are the key to improving the performance of aero engines and are suitable for the high-temperature extreme working environment of new-generation aero engines [1,2,3,4,5]. When X-rays interact with matter, amplitude attenuation occurs. Industrial computed tomography (ICT) based on amplitude attenuation was invented in the 1970s and has been widely used in the aerospace and microelectronics industries. ICT serves as a powerful analytical tool that enables the evaluation of internal structures and provides a guarantee for the detection of cermet turbine blades of new-generation aero engines [6,7,8].

ICT image reconstruction algorithms generally assume that X-ray sources emit monoenergetic rays. However, actual ray sources generally have a wide energy spectrum. Since low-energy rays are more easily absorbed by blades than high-energy rays, when X-rays penetrate the blade, the average energy of its energy spectrum becomes higher, which is called the beam hardening effect [9,10], which leads to hardening artifacts during reconstruction. The hardening artifact is exacerbated due to the material properties of cermet turbine blades. Therefore, hardening artifact correction has always been a hot topic in the field of CT application research. There are many correction methods [11,12,13,14,15]. In terms of implementation methods, they can be divided into two categories, i.e., the hardware method and the software method.

A typical hardware method is the filter pre-filtering method [16,17]. Metal materials with high attenuation characteristics are used as filters to reduce the proportion of low-energy rays. Pre-hardening is used to reduce the impact of hardening effects during the imaging process. This method is simple and easy to implement, but it leads to a reduction in the ray intensity and the signal-to-noise ratio. Its correction is not complete because it only changes the wide energy spectrum into a narrow energy spectrum.

Typical software-hardening-correction methods include the polynomial fitting method, iterative correction method, and dual-energy correction method [18,19,20]. The polynomial fitting method performs mapping correction based on the functional relationship between the projections of single-material objects before and after hardening. Kyriakou et al. [21] proposed an a priori hardening correction algorithm, i.e., Empirical Beam Hardening Correction (EBHC). The only prior knowledge of this method is to segment the reconstructed image into water and bone. A linear combination of different CT-based images for reconstruction is obtained. When the result is the flattest, it is the corrected result. The correction accuracy of this method is also affected by the binarization accuracy. The iterative correction method generally uses prior knowledge to establish a physical model and continuously approaches the true value through iterative calculations. Brabant et al. [22] proposed a beam hardening correction method based on the simultaneous algebraic reconstruction technique (SART) reconstruction algorithm, which modeled beam hardening and combined it into the forward projection of the SART reconstruction algorithm. This method can be used without a ray source energy spectrum or material property information. However, it has the disadvantages of large calculation amounts and low parallelism and is not widely used in practice. Alvarez et al. [23] proposed the dual-energy method. Through two scans of different energies, the linear attenuation coefficient distribution of the object under a certain fixed energy is determined, and then the image is hardened and corrected. This method can perform beam hardening correction on multi-material objects. The dual-energy method has relatively high hardware requirements and requires prior knowledge of the ray energy spectrum distribution.

Consider the advantages of the polynomial fitting method among software correction methods (simpleness, effectiveness, convenience, and wide usefulness). Therefore, this study researches correction methods for hardening artifacts in tomographic images of cermet turbine blades, which solves the problem of image quality degradation caused by spectral polychromaticity. Given the hardening artifacts existing in ICT imaging, this study implemented an iterative linear-fitting-based hardening artifact correction technology framework. First, an iterative binarization process based on the initial reconstructed image was conducted to improve segmentation accuracy. Second, an analysis of the selection of the fitting function model in the proposed framework was conducted. A fitting method based on the piecewise Hermite function model was derived to obtain the fitting method and iteration hyperparameters suitable for cermet turbine blades. Finally, the proposed iterative fitting method based on the Hermite function model and various polynomial fitting methods was used to fit the blade data to obtain the fitting curve. The obtained curves performed hardening correction on the blade data to obtain different groups of tomographic images. Three image-evaluation indicators were used to quantitatively analyze different groups of tomographic images. The results show that the root-mean-square error (RMSE) of the image using the proposed method is 0.0133, which is lower than that of the compared method. The peak signal-to-noise ratio (PSNR) is 37.7050 dB, and the feature structural similarity (FSIM) is 0.9881, both of which are higher than those of the compared method. Therefore, the proposed iterative linear fitting hardening artifact correction framework reduces the hardening artifacts of blade tomographic images, improves image quality, and has strong robustness, which provides new ideas for the development of the new-generation aero-engine turbine blade imaging.

## 2. Theoretical Basis and Methods

Commonly used CT reconstructions are based on monochromatic rays, i.e., the default linear attenuation is constant for a given substance. In practical applications, X-ray sources generally have a continuous energy spectrum. The same material has different attenuation coefficients under different energy rays. The attenuation coefficient corresponding to high-energy rays is smaller than that of low-energy rays, i.e., there is less attenuation across the same distance. The proportion of the high-energy part of the energy spectrum of polychromatic rays increases after penetrating the object. Therefore, this work proposes an iterative linear fitting preprocessing method. In order to explore the feasibility of the linear fitting method, this section will first explain the linear fitting principle of absorption contrast and deduce and analyze the feasibility of the linear fitting correction framework.

### 2.1. Feasibility Analysis of Linear Fitting Correction

**Figure 1:**
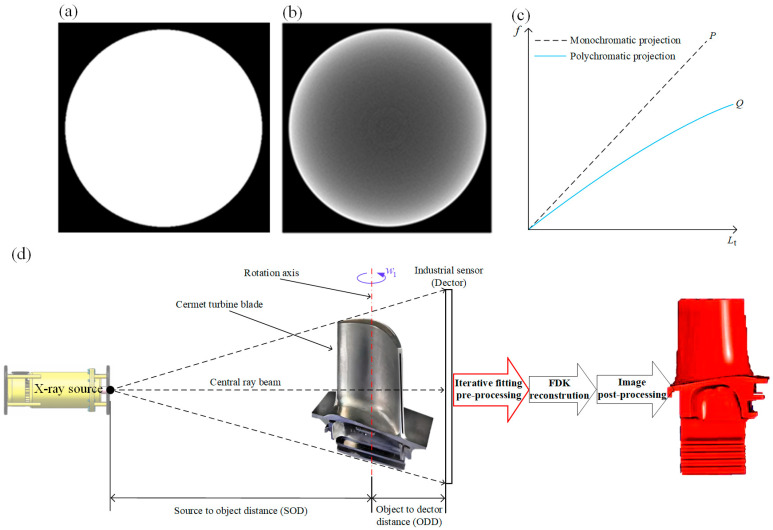
(**a**) Ideal reconstruction result; (**b**) reconstruction result with hardening artifacts; (**c**) mapping relationship between polychromatic projection Q and penetration length $L_{t}$; (**d**) the experimental ICT system configuration, and the proposed linear fitting pre-processing technology for cermet turbine blade imaging.

Hardening artifacts can cause inconsistencies between actual morphology and reconstructed images. Comparing the ideal tomographic image of the metal disc shown in Figure 1a, the tomographic image in Figure 1b is obtained by reconstructing the data collected from the metal disc without hardening correction. Degraded tomographic images are characterized by bright edges and dark centers. Since the hardening projection and penetration length satisfy a one-to-one functional relationship, the linear fitting method can be used to correct the hardening artifact. Therefore, linear fitting preprocessing based on the collected aero-engine turbine blade data is performed, and then, high-quality images can be obtained using the Feldkamp-Davis-Kress (FDK) reconstruction algorithm [24]. Figure 1d shows the configuration of the ICT system. The industrial sensor obtains data information on cermet turbine blades through X-ray full scanning. The proposed iterative fitting data preprocessing framework is performed on the sensor-collected data. The FDK reconstruction algorithm is used to obtain high-quality tomographic images after correction, which provide the basis for subsequent image post-processing.

The feasibility of linear fitting correction needs to be mathematically analyzed. Assume that the ray source defaults to a monochromatic ray. In order to obtain the attenuation coefficient distribution μ(x,y,z) of the object, the projection P of μ at each angle and position needs to be obtained. The initial intensity of the monochromatic ray is $I_{0}$, and the intensity after penetrating the object through a straight line L(θ,s) at a certain angle is I. P and μ satisfy the relationship shown in Equation (1).

(1) $$P=\ln\left(\frac{I_{0}}{I}\right)=\int_{L}^{\;}\mu (x,y,z)dL$$

According to Equation (1), P can be reconstructed to obtain the attenuation coefficient distribution μ(x,y,z). However, for actual polychromatic ray sources, their polychromatic projections Q and μ satisfy the relationship shown in Equation (2).

(2) $$Q\begin{array}{c} =\ln\left(\frac{I_{0,q}}{I_{q}}\right)=\ln\left(\frac{\int_{E}S\left(E\right)dE}{\int_{E}S\left(E\right)\exp(-\int_{L}\mu \left(x,y,z,E\right)dL)}\right)=\int_{L}\mu_{q}\left(x,y,z\right)dL \end{array}$$

where $S\left(E\right)$ represents the energy spectrum distribution, and $\mu_{q}\left(x,y,z\right)$ represents the equivalent attenuation coefficient distribution obtained as a polychromatic reconstruction. Consider that Q is not equal to the line integral of the attenuation coefficient $\mu \left(x,y,z,E\right)$ under a certain energy. Reconstructing Q can only obtain the equivalent attenuation coefficient distribution $\mu_{q}(x,y,z)$ under the voltage energy spectrum. Since $\mu \left(E\right)\sim E^{-3}Z^{3}(E<40\;\mathrm{keV})$, the attenuation coefficient μ of the low-energy component of the ray is larger, and the average energy of the ray after penetrating the object will become higher. Take the cermet turbine blade as an example, the thicker the ray passes through the blade, the more obvious this hardening effect is. The $\mu_{q}$ tends to be generated by high-energy rays and becomes smaller. Therefore, cupping artifacts are generated in thicker parts of the blade.

When the energy spectrum of the ray source and the material distribution are unknown, it is not feasible to obtain the attenuation coefficient distribution μ(x,y,z,E). But when the object to be inspected is of a single material, Equation (2) can be specialized into Equation (3).

(3) $$Q=-\ln\left(\int_{E}\omega \left(E\right)exp\left(-\mu \left(E\right)L_{t}\right)dE\right)=\int_{L_{t}}\mu_{q}\left(x,y,z\right)dL_{t}$$

In Equation (3), $\omega \left(E\right)=S\left(E\right)/\int_{E}S(E)dE$ represents the energy proportion of each energy spectrum. $L_{t}$ represents the thickness of the ray penetrating the object. When the energy spectrum of the X-ray source $\omega \left(E\right)$ is determined, Q and $L_{t}$ form a functional relationship f, as shown in Equation (4).

(4) $$Q=f\left(L_{t}\right)$$

In order to observe the trend of the curve, the derivative f′ of the function f is obtained according to Equation (3), as shown in Equation (5).

(5) $$f^{\prime }\left(L_{t}\right)=Q^{\prime }\left(L_{t}\right)=\frac{\int_{E}\omega \left(E\right)\mu \left(E\right)exp\left(-\mu \left(E\right)L_{t}\right)dE}{\int_{E}\omega \left(E\right)exp\left(-\mu \left(E\right)L_{t}\right)dE}=\int_{E}\omega_{q}\left(E,L_{t}\right)\mu \left(E\right)dE$$

In Equation (5), $\omega_{q}\left(E,L_{t}\right)=I_{q,E}\left(E,L_{t}\right)/I_{q}(L_{t})$ represents the energy proportion of each ray after penetrating the length $L_{t}$. It can be deduced that $f\prime \left(L_{t}\right)$ is the weighted value of the attenuation coefficient of each energy penetrating the object. The larger the penetration length $L_{t}$, the more obvious the hardening. In Equation (5), μ(E) tends to the high energy. Therefore, $f\prime (L_{t})$ is a monotonically decreasing function, and $f(L_{t})$ is a concave function about $L_{t}$. The mapping relationship between the polychromatic projection Q and penetration length $L_{t}$ is shown in Figure 1c.

This is since the penetration length $L_{t}$ and the projection Q have a one-to-one corresponding functional relationship f. In engineering, the linear fitting method can be used to obtain the functional relationship f, and then, $Q\left(\theta ,s\right)$ is mapped to $\tilde{L_{t}}\left(\theta ,s\right)$, as shown in Equation (6).

(6) $$\tilde{L_{t}}\left(\theta ,s\right)=f^{-1}\left(Q\left(\theta ,s\right)\right)=\int_{L}\rho \left(x,y,z\right)dL$$

According to $\tilde{L_{t}}$, the object distribution ρ(x,y,z) can be reconstructed. The equivalent attenuation coefficient of the object can be regarded as the attenuation coefficient $\mu_{p,0}$ when the penetration length $L_{t}$ approaches 0. Equation (5) can be derived to obtain Equation (7).

(7) $$\mu_{q,0}=\underset{L_{t}\to 0}{\lim}\frac{Q}{L_{t}}=\int_{E}\omega \left(E\right)\mu \left(E\right)dE$$

Therefore, the linear fitting correction feasibility analysis of the turbine blade is completed.

### 2.2. Framework Overview

This section proposes a technical framework of the iterative linear fitting correction suitable for turbine blade imaging and elaborates on the framework. The framework only needs uncorrected tomographic images to establish the functional curve relationship of the hardening correction model and map the original projection based on the functional curve to achieve the correction of hardening artifacts.

**Figure 2:**
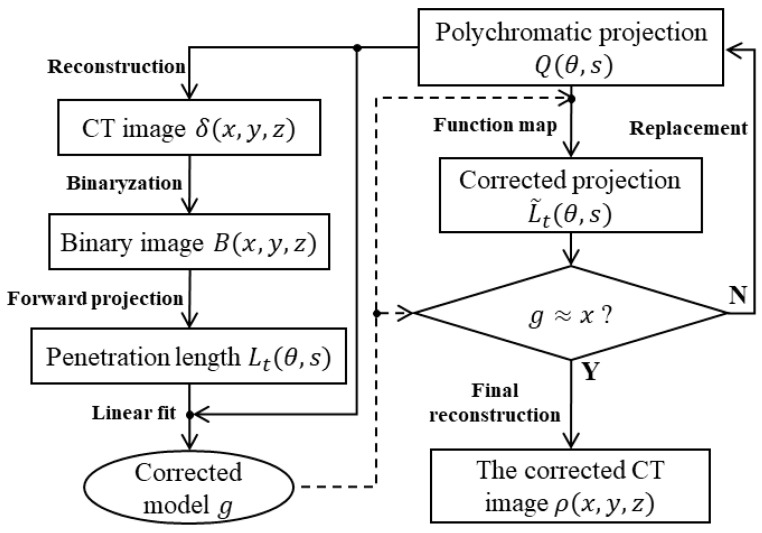
Fitting correction algorithm data flow chart.

The principle of the hardening correction technical framework and the flow chart are shown in Figure 2, in which the rectangular box represents the image data and the solid line with arrows represents the flow direction of the image data. The basic idea of the framework is to obtain the original projection Q and penetration length $L_{t}$ at each angle under cone beam imaging. Then, Q and $L_{t}$ are fitted to obtain the correction function model g (g is the inverse function of f, i.e., $g=f^{-1}$). The original projections are corrected based on the correction function model and then are reconstructed to obtain the three-dimensional (3D) object distribution coefficient ρ(x,y,z). Due to problems, such as binarization error, fitting accuracy, and projection data noise, the correction is generally performed more than once. During iteration, the original projection Q needs to be replaced by the projection ${\tilde{L}}_{t}$ that has not been completely corrected. After several iterations, if the fitting curve approximation is to a straight line, the iterative correction is completed.

The steps for the above iterative linear fitting framework to correct hardening artifacts are below.

(1) The projection information from the line integral data of the industrial sensor (detector) is obtained. Then, the projection Q after performing logarithmic demodulation is obtained. They are shown in Equation (8).

(8) $$Q=\int_{L}\rho (x,y,z)\cdot dL$$

If Q is replaced by the corrected projection ${\tilde{L}}_{t}$ in the subsequent iteration process, the magnitude of the projection Q will change to represent the entire image pixel length.

(2) FDK reconstruction is performed on the projection Q to obtain the initial uncorrected tomographic image δ(x,y,z). In the subsequent iteration process, the attenuation coefficient distribution δ(x,y,z) is finally replaced by the object distribution coefficient ρ(x,y,z). However, the distribution ρ and the binary image B are different. ρ can reflect the details and noise of the real tomographic information of the object. It is the corrected object attenuation coefficient distribution, rather than simple binarization.

(3) The OTSU threshold segmentation algorithm [25] on the uncorrected image δ(x,y,z) is performed to obtain the B(x,y,z) binary image. Due to the noise in the original projections, the binary image may have noise, which can be eliminated through binarization processing. Since hardening artifacts can lead to suboptimal segmentation, the binary map results can be updated in iterations to the effective segmentation.

(4) After the effective segmentation result B(x,y,z) is obtained, forward projection is performed on B(x,y,z) to obtain the penetration length $L_{t}(\theta ,s)$.

(5) The projection data Q and the penetration length $L_{t}$ are linearly fitted to obtain the correction model g that satisfies $L_{t}=g(Q)$. In the initial iteration, $\left(Q,L_{t}\right)$ is directly used as the data to perform the linear fit. As mentioned in step (1), during the second iteration, the projection data Q are replaced by ${\tilde{L}}_{t}$. The data are updated to $\left({\tilde{L}}_{t},L_{t}\right)$. If the hardening artifact has been completely corrected during the iteration process, the fitting curve satisfies $g\left(x\right)\approx x$, i.e., the corrected projection is basically equal to the thickness of the ray penetrating the object.

(6) The correction model g is used to correct Q to obtain the corrected projection ${\tilde{L}}_{t}=g\left(Q\right)$. In the first iteration, due to problems, such as binarization accuracy and fitting accuracy, only the preliminary correction function $g_{0}$ can be obtained. If it is a subsequent iterative process, assuming that the fitting function at the i-th iteration is $g_{i}$, the number of termination iterations is ite. The corrected projection obtained according to the final correction model is Equation (9).

(9) $${\tilde{L}}_{t,final}=g_{final}(Q)=g_{ite}\left(g_{ite-1}\left(\ldots g_{1}\left(g_{0}(Q)\right)\ldots \right)\right)$$

It can be deduced that based on the idea of fitting residuals, the final correction function $g_{final}$ is the nesting of all iterative fitting functions $g_{i}$. Projections under the same experimental conditions can be corrected using this model.

(7) It can be deduced from step (5) that if $g_{ite}(x)\approx x$, the iteration is terminated. The FDK reconstruction algorithm is performed on projection ${\tilde{L}}_{t}$ to obtain the 3D object distribution coefficient distribution ρ(x,y,z). Otherwise, Q is replaced by ${\tilde{L}}_{t}$ in step (2) to proceed to the next iteration.

In the traditional linear fitting framework, only a single binarization is performed, and the ideal binary result cannot be obtained due to severe hardening. However, the above framework in this work uses an iterative method to re-threshold the tomographic image so as to obtain better segmentation. The relevant analysis is conducted below.

## 3. Results and Discussion

### 3.1. Iterative Binarization Processing

**Figure 3:**
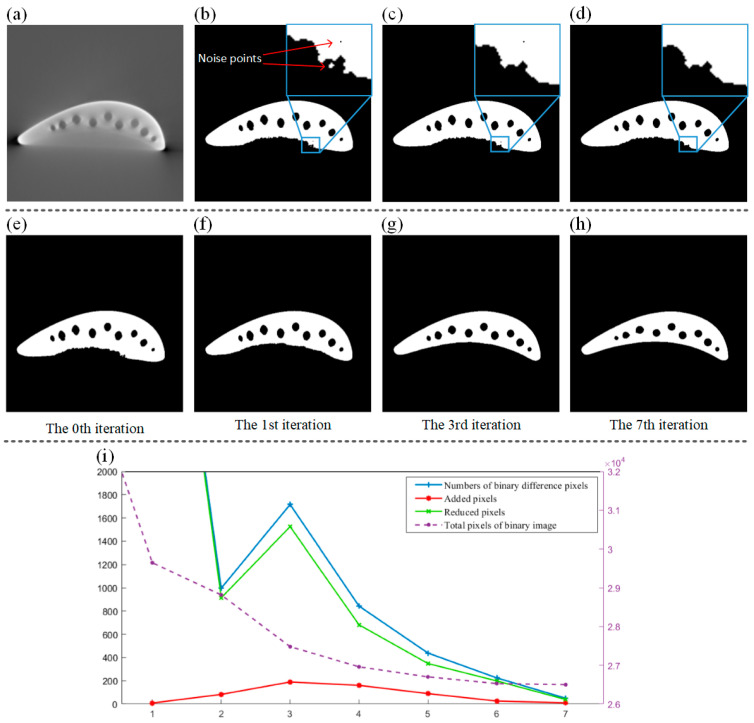
(**a**) Initial CT image without correction of artifacts; (**b**) initial binarized image; (**c**) binarized image opening operation; (**d**) binarized image closing operation; (**e**–**h**) iterative optimization of threshold segmentation of tomographic images; (**i**) attribute curves of binary segmentation results and number of iterations.

When performing threshold segmentation on the image $\delta \left(x,y,z\right),$ as shown in Figure 3a, due to the noise in the original projection, the binary image may have noise. Two operations can be carried out to remove it: ➀ binarization pre-processing, i.e., performing smoothing filtering on the projections before reconstruction and the tomographic images after reconstruction. The reconstruction results are only used for binarization, otherwise it will affect the subsequent fitting model. ➁ Binarization post-processing, i.e., after obtaining the preliminary binary image B(x,y,z), two morphological operations are performed on B(x,y,z). As shown in Figure 3c, the open operation is used to remove white noise outside the blade in B(x,y,z). As shown in Figure 3d, the closed operation is used to remove black noise inside the blade in B(x,y,z). It can be deduced that the noise in Figure 3b and the sharp edges and corners caused by segmentation have been better eliminated, which is more consistent with the original characteristics of the blade imaging.

Since the hardening artifacts of the blade are severe, the initial segmentation result is not ideal. Therefore, in each iteration, binary segmentation is performed on the reconstructed image corrected in the previous step, and the existing binary image B(x,y,z) is replaced. Set up the pixel similarity threshold $T_{B}$. When the number of different pixels between the binary image B(i) generated by the i-th iteration and the previous binary image B(i−1) is less than $T_{B}$, as shown in Equation (10), it can be considered that the segmentation effect has reached the optimal level.

(10) $$\sum_{m,n}\left|B\left(i\right)-B\left(i-1\right)\right|<T_{B}$$

where (m,n) are the 2D coordinates of the image pixels. Subsequent iterations will no longer update the binary image B(x,y,z).

As shown in Figure 3e–h, the binary segmentation results gradually approach the profile of the turbine blade as the number of iterations increases. It can be seen that the difference in results decreases with the iterations. As shown in Figure 3i, curves of the number of the binary difference pixels, the number of added and reduced pixels, and the total pixels of the binary image with respect to the number of iterations are drawn. It can be deduced that as the number of iterations increases, the binary difference area steadily decreases, the quality of the reconstructed image gradually becomes stable, and the total area of the binary image converges to a constant value, i.e., the final binary result.

### 3.2. Fitting Function Selection

In the proposed linear fitting technology framework, the selection of the fitting function model is crucial, which directly influences the accuracy of the fitting curve and the final correction effect. This section summarizes the polynomial function model of traditional linear fitting and analyzes its disadvantages. Therefore, this section proposes a new fitting model combined with the Hermite curve [26] to improve the shortcomings of the polynomial function to achieve better correction effects.

When the projection data Q and the penetration length $L_{t}$ are linearly fitted, the existing linear correction method generally uses the polynomial fitting method, which assumes that the correction function g is a polynomial, as shown in Equation (11).

(11) $$g\left(Q,\omega \right)=\omega_{n}Q^{n}+\omega_{n-1}Q^{n-1}+\cdots +\omega_{1}Q+\omega_{0}=\sum_{i=0}^{n}\omega_{i}Q^{i}$$

where n is the polynomial order, the vector ω represents $\left(\omega_{0},\omega_{1},\ldots ,\omega_{n}\right)$, and the maximum likelihood $\omega_{ML}$ is Equation (12).

(12) $$\omega_{ML}=arg\underset{\omega }{\min}\sum_{\left(Q,L_{t}\right)}{\left(g\left(Q,\omega \right)-L_{t}\right)}^{2}$$

Substituting this $\omega_{ML}$ into Equation (11), the polynomial function relationship $g(Q,\omega_{ML})$ satisfied by the data $\left(Q,L_{t}\right)$ is obtained.

**Figure 4:**
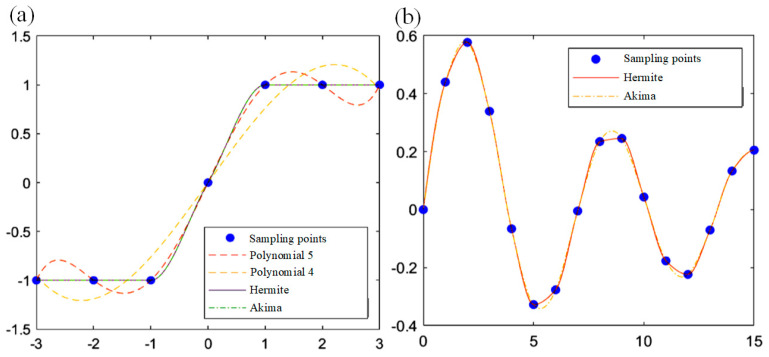
(**a**) Fitting effect curves of different fitting methods; (**b**) fitting effect comparison curve of Akima and Hermite.

The mathematical characteristics of polynomials determine that when the order n is high, it is easy to overfit in functions. When the order n is reduced, local details cannot be well fitted. An example can be used to explain the characteristics of polynomial fitting. As shown in Figure 4a, seven sampling points equally spaced in the (−3≤x≤3) are fitted in different ways. It can be observed that polynomial 5 fittings can pass through all sampling points (equivalent to interpolation), but its fitting curve is too steep at the corner of the sampling point of x=1, resulting in an over-fitting phenomenon of up and down fluctuations. The polynomial 4 fitting can better reflect the overall trend of the sampling points, but it cannot reflect the details at x=1. Piecewise Akima interpolation [27] and piecewise Hermite interpolation have similar fitting effects. It can avoid “overshooting” the curve at the corners and accurately connect the platform areas, making up for the shortcomings of the polynomial fitting. To further observe the difference in characteristics between the two piecewise interpolation functions, the sampling points to be fitted in Figure 4b are generated by the oscillation sampling function. Akima interpolation can better capture the fluctuations between points, while Hermite interpolation sharply flattens at local extrema.

Based on the above analysis, this work proposes a fitting framework based on Hermite interpolation to improve the accuracy of curve fitting. The iterative fitting framework adopted in this work has higher requirements for precision than traditional single fitting. The correction of the initial curve to be fitted is the same as the traditional single linear correction, the curve that needs to be fitted later is the residual curve that was not fully corrected in the previous time. The residual curve is getting closer to the target straight line y=x as the number of iterations increases. The use of Hermite interpolation can avoid the disadvantages of polynomial fitting, i.e., a sharp inflection point at the end of the fitting curve that does not meet the expectations, and the subsequent correction will use the wrong fitting curve, resulting in serious over calibrated artifacts of the mapping projection, which will affect the reprojection and fitting of subsequent iterations and make the curve unable to converge to get the best correction curve.

To make Hermite interpolation more suitable to the iterative fitting framework, the Hermite interpolation is optimized. The Hermite interpolation function satisfies the requirement that the function at the node and the first derivative of the function are equal to the relevant value. If there are (n+1) mutually different nodes $a\leq x_{0},x_{1},x_{2},\ldots ,x_{n}\leq b$, and their corresponding function values are $y_{0},y_{1},y_{2},\ldots ,y_{n}$, then, there can be a Hermite function H(x) that satisfies Equations (13) and (14).

(13) $$H\left(x_{i}\right)=y_{i},i=0,1,\ldots ,n$$

(14) $$H^{\prime }\left(x_{i}\right)=y_{i}^{\prime },i=0,1,\ldots ,n$$

Based on this, the expression $H\left(x,x_{r},y_{r}\right)$ of $H\left(x\right)$ can be solved, where the vector $x_{r}=\left(x_{0},x_{1},\ldots ,x_{n}\right)$ and the vector $y_{r}=\left(y_{0},y_{1},\ldots ,y_{n}\right)$. If this function is used to represent the functional relationship of the correction model, the correction function g can be expressed as Equation (15).

(15) $$g\left(Q,x_{r},y_{r}\right)=H\left(Q,x_{r},y_{r}\right)$$

Theoretically, the least squares method can be used directly to obtain the maximum likelihood $x_{r,ML}$ and $y_{r,ML}$, as shown in Equation (16).

(16) $$\begin{array}{c} \left(x_{r,ML},y_{r,ML}\right)=\arg\underset{\left(x_{r},y_{r}\right)}{\min}\sum_{\left(Q,L_{t}\right)}{\left(g\left(Q,x_{r},y_{r}\right)-L_{t}\right)}^{2} \end{array}$$

**Figure 5:**
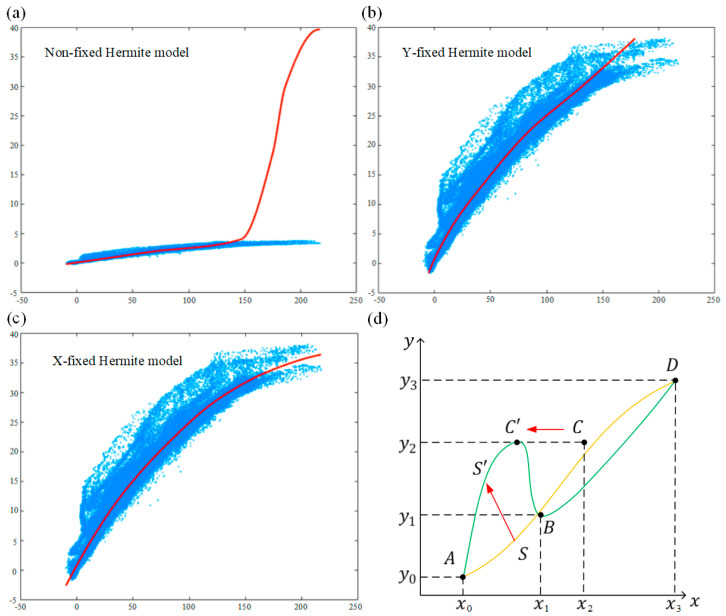
The optimal configuration scheme of Hermite fitting is selected. (**a**) Unfixed Hermite fitting; (**b**) Hermite fitting with a Y-fixed direction; (**c**) Hermite fitting with an X-fixed direction; (**d**) the discontinuity of L concerning $x_{r}$ when fixed $y_{r,0}$.

However, in the actual process of iteratively calculating the minimum value, since $\left(x_{r,ML},y_{r,ML}\right)$ has a total of (2n+2) variables that need to be solved, the optimal solution cannot be obtained within a limited time. As shown in Figure 5a, blue area are points to be fitted, red line is the fitted curve. $x_{r}$ is set to a fixed value $x_{r,0}$ uniformly distributed in the horizontal axis direction under this condition. Its expression is Equation (17).

(17) $$x_{r,0}\left(i\right)=x_{i}=Q_{min}+\frac{\left(Q_{max}-Q_{min}\right)}{n}\cdot i$$

where $Q_{min}$ and $Q_{max}$ are the maximum value and minimum value of the projection respectively. Therefore, Equation (16) is transformed into Equation (18).

(18) $$y_{r,ML}=arg\underset{y_{r}}{\min}\sum_{\left(Q,L_{t}\right)}{\left(g\left(Q,x_{r,0},y_{r}\right)-L_{t}\right)}^{2}$$

Substituting $y_{r,ML}$ into Equation (15) to get the functional relationship $g(Q,x_{r,0},y_{r,ML})$ of the data $\left(Q,L_{t}\right)$ is satisfied. In the same way, $y_{r}$ can also be set to a fixed value $y_{r,0}$ uniformly distributed in the vertical axis direction, so that Equation (16) can be converted into Equation (19).

(19) $$x_{r,ML}=arg\underset{x_{r}}{\min}\sum_{\left(Q,L_{t}\right)}{\left(g\left(Q,x_{r},y_{r,0}\right)-L_{t}\right)}^{2}$$

When $x_{r,ML}$ is substituted into Equation (15) to obtain the functional relationship $g(Q,x_{r,ML},y_{r,0})$ which is satisfied by data $\left(Q,L_{t}\right)$.

To verify the feasibility of the above derivation, the experimental data were fitted. Due to the presence of noise and the fact that the binary map is not accurate enough without iteration, its projection data are affected, resulting in a “bifurcation” at the right end of the points to be fitted. This situation is common and tests the selection of the fitting algorithm. Once the robustness of the fitting algorithm is not strong enough, it is easy to overfit or underfit, which may cause the consequences of residual fit failure during subsequent iterations. The default Hermite interpolation fitting, fixed $y_{r,0}$ and fixed $x_{r,0}$ were used for fitting respectively, and the effects are shown in Figure 5b,c. It can be deduced that selecting a fixed $x_{r,0}$ or $y_{r,0}$ before using the optimization to obtain the maximum likelihood value of $\left(x_{r},y_{r}\right)$ can greatly improve the accuracy of the fitting. It will be more accurate in the horizontal axis direction.

The above shows that it is easier to converge to the best fitting node by uniformly fixing the horizontal axis node than the vertical axis. This is because the target curve to be fitted is a function $g_{0}(x)$ about the horizontal axis. Let the target term in Equation (16) be L, which is Equation (20).

(20) $$L=\sum_{\left(Q,L_{t}\right)}{\left(g\left(Q,x_{r},y_{r}\right)-L_{t}\right)}^{2}$$

Mathematically, it is easy to find that small changes in any component of $y_{r}$ after fixing $x_{r,0}$ will only cause continuous changes in g(x). The same is true for L, so L is continuously differentiable concerning $y_{r}$. After fixing $y_{r,0}$, the continuous change in a certain component of $x_{r}$ may cause the function L to mutate. As shown in Figure 5d, the ordinates of the four nodes A,B,C,D uniformly fixed in the *y*-axis direction are $y_{r,0}=\left(y_{0},y_{1},y_{2},y_{3}\right)$. At a certain position $x_{r}$, the fitting curve is g(x)=S. It is assumed that C is infinitely close to B in the horizontal axis direction, i.e., $x_{2}\to {x_{1}}^{+}$. If the abscissa of C changes slightly and moves in the negative direction of the horizontal axis to C′, i.e., $x_{2}\to {x_{1}}^{-}$, the new fitting curve S′ changes suddenly relative to the original curve S. According to Equation (20), it can be deduced that the corresponding objective function L will also mutate. Therefore, L is discontinuous concerning $x_{r}$. When the objective function has discontinuous regions, it will have a greater impact on the optimization results, so the fixed $x_{r,0}$ horizontal axis coordinate is finally selected to obtain higher fitting accuracy.

**Figure 6:**
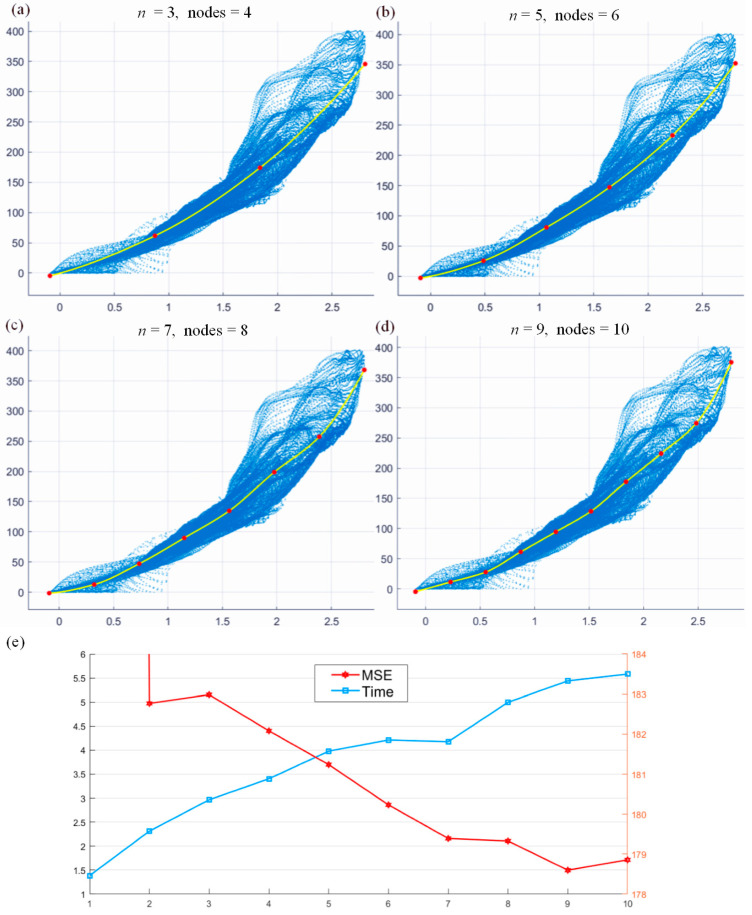
(**a**–**d**) Hermite fitting of different nodes; (**e**) MSE and time relationship curves concerning *n*.

Similar to polynomial fitting, the proposed Hermite fitting method in this work also has a unique hyperparameter n, which is used to control the number of nodes (n+1) in H(x). The more nodes there are, the higher the precision of the fitting, but it is also easier to overfit. Different n values are selected for fitting the same set of experimental data points, and the fitting effect is shown in Figure 6. As shown in Figure 6a, when n=3, due to the small number of fitting nodes (red points), the interval x=0.2~0.8 close to the origin is not fully fitted. As shown in Figure 6b–d, when the n value is increased, i.e., n≥5, the problem has been solved. However, Figure 6c,d cause overfitting due to the large n value. Its derivative is non-monotonous and fluctuates up and down, which does not conform to the original overall characteristics of the curve. If iterative fitting is performed using this result, it will inevitably have adverse results.

To quantitatively analyze the impact of different *n* values on the fitting accuracy, the mean square error (MSE) and time of n=1~10 were statistically plotted as shown in Table 1 and Figure 6e. The MSE is defined here as Equation (21).

(21) $$MSE=\frac{L_{ML}}{N}=\frac{\sum_{\left(Q,L_{t}\right)}{\left(g\left(Q,x_{r,0},y_{r,ML}\right)-L_{t}\right)}^{2}}{N}$$

MSE represents the residual deviation between the fitting curve and the points to be fitted. As shown in Figure 6e, since the Hermite curve degenerates into a linear function, the MSE is extremely large when n=1. As the value n increases, the MSE of the fitted curve gradually decreases, but the calculation time also increases. Since the optimization problem itself does not guarantee convergence to the optimal solution in a stable time, MSE has an upward reverse trend when n=3 and n=10, and the calculation time when n=7 is also smaller than when n=6. When n=4~5, the MSE after fitting is already small. Therefore, it will not cause over-fitting to the experimental data at the same time, which can better reflect the overall trend in the data points. Therefore, n=5 is selected as the ideal hyperparameter in subsequent Hermite fittings.

### 3.3. Fitting Effect Verification

To verify the superiority of the proposed Hermite fitting pre-processing method compared with the other methods, the polynomial 6 fitting, polynomial 5 fitting, the polynomial 4 fitting, polynomial 3 fitting, and the Akima fitting were used to perform linear correction on the experimental data of aero-engine blades, and the final results were compared with those of Hermite fitting method. First, we need to analyze the fitting curves of the blade data and observe that the curves reach the optimal iterations applicable to the blade data. Finally, we used the above methods to preprocess the uncorrected projections to get the final tomographic images.

#### 3.3.1. Analysis of Different Iterative Fitting Curves

**Figure 7:**
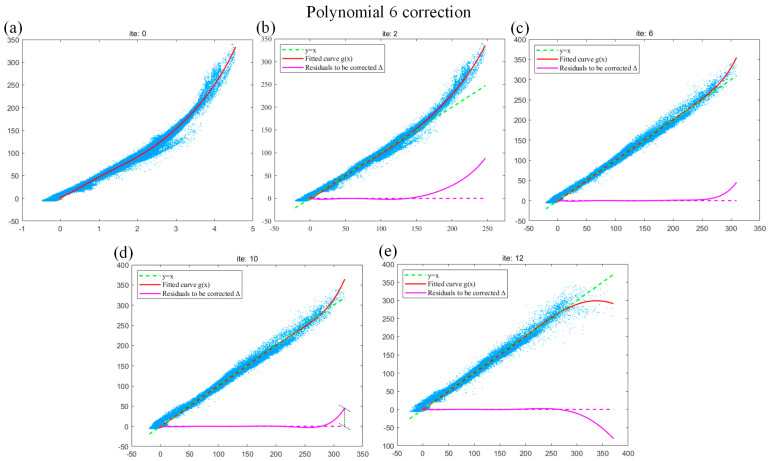
(**a**–**e**) The fitting effects of the 1st, 2nd, 6th, 10th, and 12th iterations of the polynomial 6.

As shown in Figure 7, we use the blade data to analyze the iterative fitting curve of the commonly used polynomial 6 and obtain the optimal number of iterations when the curve reaches convergence. We observe the relevant fitting curve of different iterations. As shown in Figure 7a, when the polynomial 6 completes the initial fitting, it can be observed that the curve is smooth and can better reflect the trend of data. As shown in Figure 7b, when the polynomial 6 is fitted for the second time, the horizontal axis data Q in the second iteration is replaced with the corrected projection length ${\tilde{L}}_{t}$, so that the fitting curve can be used for the subsequent correction of residuals (the green dashed line represents the iteration termination line y=x, and the pink solid line represents the current uncorrected residual ∆=g(x)−x). It can be deduced that the polynomial fitting effect of this iteration is also better, but the curve is far from y=x. As shown in Figure 7c, when the polynomial 6 is fitted for the sixth time, the fitting curve continues to approximate y=x and is well fitted with the data points. As shown in Figure 7d, when the polynomial 6 is fitted for the 10th time, the fitting curve fits the data points well and is close to y=x, but overfitting occurs at the right endpoint of the fitting curve. As shown in Figure 7e, if the iteration continues, the overfitting reverse deviation of the right endpoint of the fitting curve becomes more serious when the polynomial 6 is fitted for the 12th time. This is the disadvantage of polynomial fitting mentioned above, i.e., overfitting is easy to occur when the order is too high. In summary, for the blade data, the polynomial 6 can obtain relatively good results at the 10th iteration.

**Figure 8:**
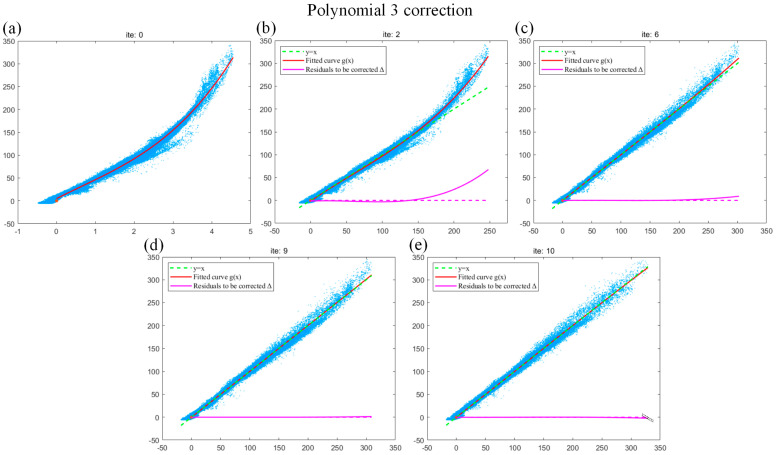
(**a**–**e**) The fitting effects of the 1st, 2nd, 6th, 9th, and 10th iterations of the polynomial 3.

Then, we use the blade data to analyze the iterative fitting curve of the polynomial 3 and obtain the optimal number of iterations when the curve reaches convergence. We observe the relevant fitting curve of different iterations. As shown in Figure 8a, when the polynomial 3 completes the initial fitting, it can be observed that the curve is smooth overall, but the data trend is abnormal at the right endpoint. As shown in Figure 7b, when the polynomial 3 is fitted for the second time, it can be observed that the fitting effect of the iterative polynomial is better, but is far from y=x. As shown in Figure 7c,d, when the polynomial 3 is fitted to the 6th and 9th degrees, the corresponding data trend is poor even though the fitting curve is close to the right endpoint of y=x. This is the disadvantage of polynomial 3 fitting because underfitting easily occurs when the order is low. As shown in Figure 7e, when the polynomial 3 is fitted for the 10th time, the fitting curve is close to y=x and the fitting effect of the right endpoint is better than that of Figure 7c,d. In summary, for the blade data, the polynomial 3 can obtain relatively good results at the 10th iteration.

**Figure 9:**
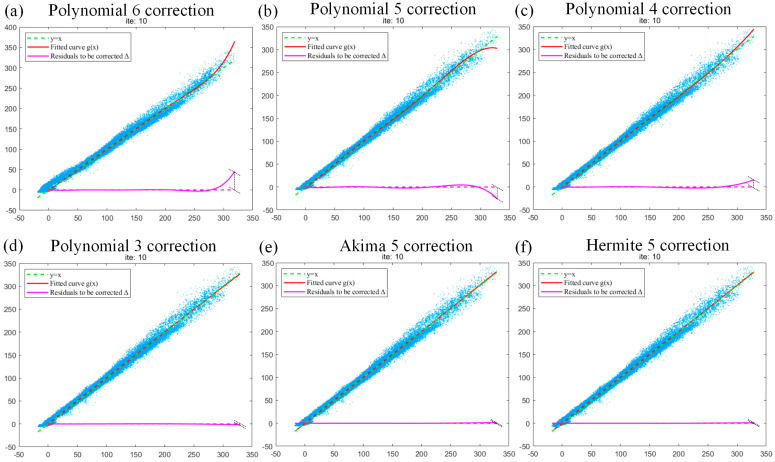
(**a**–**f**) The fitting curves of the optimal number of iterations of polynomial 6, polynomial 5, polynomial 4, polynomial 3, Akima 5, and Hermite 5, respectively.

Through the above iterative optimization analysis of fitting curves for the polynomial 6 and the polynomial 3, it can be deduced that the optimal number of iterations can be obtained only when overfitting and underfitting phenomena do not occur and the fitting curve approximates y=x in the fitting process of the blade data. Therefore, on this basis, we used the polynomial 5 and the polynomial 4, respectively, to analyze the fitting curve for the blade data to determine that the optimal number of iterations is 10. As shown in Figure 9, by comparing the fitting curves of polynomial 6, polynomial 5, polynomial 4, polynomial 3, Akima 5, and Hermite 5, it can be observed that the fitting effect of polynomial 5 (Figure 9b) is similar to that of polynomial 6 (Figure 9a), i.e., the serious overfitting phenomenon exists in polynomial 5. Overfitting in polynomial 4 is relatively mild. Although polynomial 3 has no overfitting phenomenon, if polynomial 3 is used in the initial iteration, the iteration will be prematurely terminated due to its insufficient fitting accuracy. At the end of the iteration, the end of the fitted curve has an underfitting effect, which will still cause the residual hardening of the final image. Akima 5 and Hermite 5 fitting curve analyses are performed to observe the same optimal number of iterations. The fitting effect of Akima 5 and the proposed Hermite 5 in this work is shown in Figure 9d–f, which can inherit the advantages of polynomial fitting and make up for the disadvantages of overfitting. In the final iteration, the uncorrected residual curve approaches 0.

As for the disadvantages of polynomial fitting, there are two prospects for optimization in the future. For example 1, setting a threshold *N* and using polynomial 6 to fit in the first *N* iterations and then using polynomial 3 can be considered. However, it introduces a new threshold *N*. Due to the fact that the number of iterations required to obtain the final correction model is different for different data, the effect of using the same threshold cannot perfectly balance the fitting accuracy and convergence of various experimental data. For example 2, it is also considered to add regular terms to polynomial 6 to avoid overfitting, i.e., to add $\lambda {\left\|\omega \right\|}^{2}$ terms (λ is the regular coefficient) to Equation (12). This will introduce new challenges: ➀ it is difficult to determine the proper regular coefficient λ; ➁ although $\omega_{ML}$ has a unique solution, due to the matrix inversion operation involved, when the data scale is large, the matrix inversion speed is slow. However, for the experimental data with a large number of points and nonuniform distribution in the direction of the horizontal axis, the use of high-order polynomials and gradient-based optimization methods sometimes leads to solution failure. Therefore, the proposed Hermite 5 fitting method has good applicability at present.

Akima 5 and the proposed Hermite 5 in this work have been relatively applicable to the present experimental data. The fitting effect can not only inherit the advantages of polynomial fitting, but also make up for the disadvantages of overfitting, and the uncorrected residual curve approaches 0 in the final iteration. As for the quality evaluation of the final tomographic image, we discuss it in Section 3.3.2.

#### 3.3.2. Tomographic Images of Different Iteratively Fitting Methods and Their Evaluations

**Figure 10:**
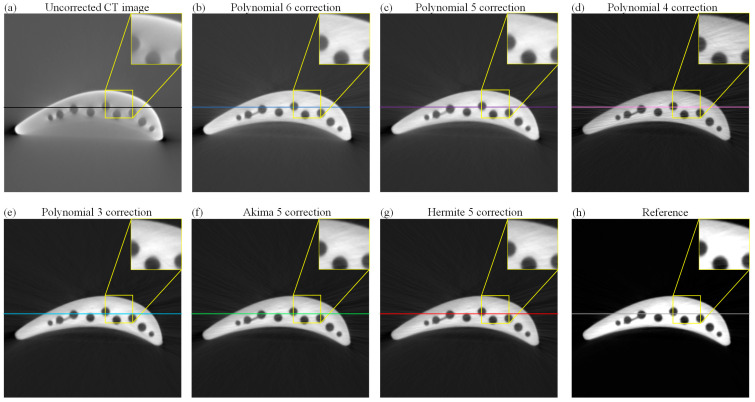
(**a**) Initial tomographic image without the correction of hardening artifacts; (**b**–**g**) tomographic images after correction using different fitting methods; (**h**) reference tomographic image.

After obtaining the fitted curve of different methods, in order to verify the consistency of the curve-fitting effect and their corresponding final reconstructed tomographic images, we reconstructed the uncorrected projections and the corrected projections using the above algorithms, respectively, as shown in Figure 10. It can be deduced that the tomographic images of the uncorrected projection in Figure 10a have severe hardening artifacts, with shadows on the inside of the object’s ellipse-like structure and dark strip artifacts at the corners. Figure 10h shows the high-quality tomographic image (reference) obtained via complex processing. Figure 10b–g shows the tomographic images obtained using the corrected projection reconstruction of several algorithms. Compared with the uncorrected tomographic image in Figure 10a, visual observation showed that other corrected images were successively improved and gradually approached the reference shown in Figure 10h. However, through an analysis of the advantages and disadvantages of the above polynomial algorithm, the tomographic images reconstructed using the polynomial 6, 5, and 4 correction, as shown in Figure 10b–d, result in the residual hardening artifacts due to the overfitting effect. In Figure 10e, the tomographic image reconstructed based on the polynomial 3 correction projection makes some details in the image unclear due to the overcoincidence effect. Akima 5 and Hermite 5 algorithms in Figure 10f,g corrected projection-reconstructed tomographic images with high quality, which is close to the reference image.

**Figure 11:**
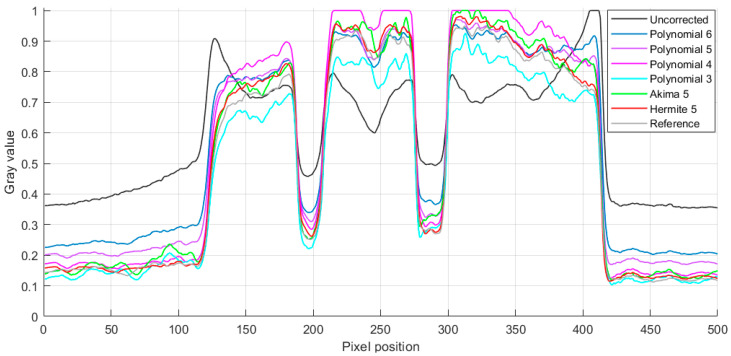
Gray-value curves along different color solid lines in Figure 10a–h.

**Figure 12:**
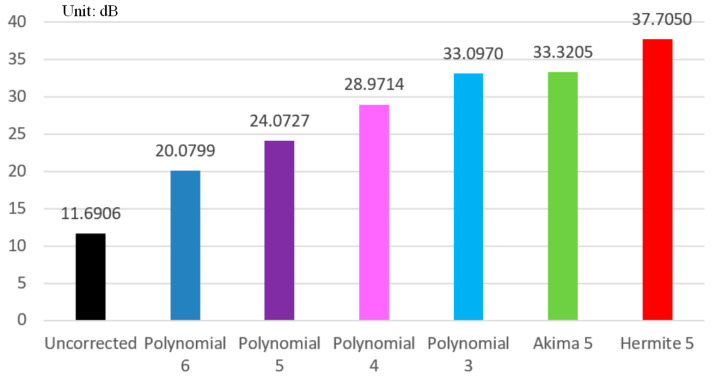
PSNR bar chart of images in Figure 10a–g.

**Figure 13:**
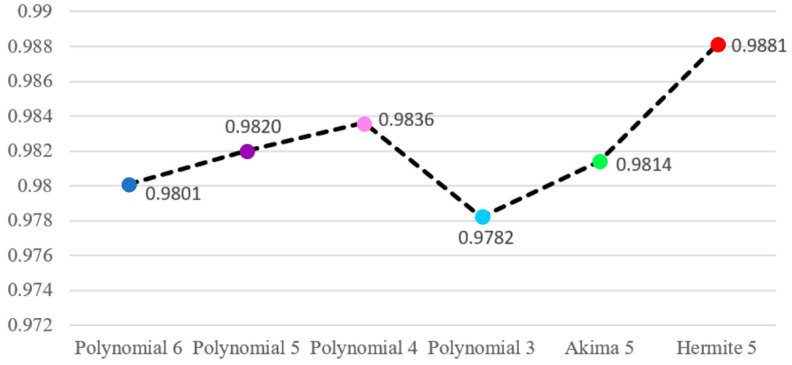
FSIM line chart of corrected images in Figure 10b–g.

For a more detailed analysis, gray-value curves were drawn on lines of different colors at the same position in Figure 10a–h to further compare the correction quality of various fitting methods. Figure 11 shows the gray-value curve of the uncorrected tomographic image, polynomial 6-corrected tomographic reconstruction, polynomial 5-corrected tomographic reconstruction, polynomial 4-corrected tomographic reconstruction, polynomial 3-corrected tomographic reconstruction, Akima 5-corrected tomographic reconstruction, and the proposed Hermite 5-corrected tomographic reconstruction. It can be observed that due to the phenomenon of overfitting or underfitting, the polynomial correction will always have a distortion on some peaks or double peaks showing high on both sides and low in the middle, which leads to the phenomenon of profile blurring or incomplete corrected hardening artifacts of the images. The gray-value curves of Hermite 5 and Akima 5 have better correction effects than other methods and are close to the reference image. In order to quantitatively evaluate image quality, the RMSE (obtain relevant information in Equation (A1) in Appendix A), PSNR (obtain relevant information in Equation (A2) in Appendix A), and FSIM (obtain relevant information in Equation (A3) in Appendix A) are calculated for the correction results of these six different fitting methods, respectively, as shown in Table 2 and Figure 12 and Figure 13. The RMSE 0.0133 calculated using Hermite 5 is the smallest among the other correction methods, which reflects the smallest difference between the tomographic reconstruction corrected by Hermite 5 and the reference image. The calculated PSNR 37.7050 dB and FSIM 0.9881 of Hermite 5 are the largest among all correction methods, which shows that the tomographic reconstruction of the Hermite 5 correction is the closest to the reference image. By observing the bar chart of PSNR shown in Figure 12 and the line chart of FSIM shown in Figure 13, we can see that the Hermite 5 correction method has strong robustness and will not show the imbalance of the quantitative evaluation results of the other methods. Therefore, through three quantitative evaluation indicators of image quality, we concluded that the quantitative analysis is consistent with the visual observation. The Hermite 5 fitting method can promote the image quality of blade tomographic images and provide a new idea for next-generation aero-engine turbine blade imaging.

## 4. Conclusions

Based on the research background of high-quality imaging of new-generation aero-engine turbine blades, to solve the problem of ICT image quality degradation caused by the energy spectrum polychromism of cermet turbine blades, an ICT hardening artifact correction technical framework based on iterative linear fitting is proposed in this work. First, the proposed framework uses a continuous iterative binarization method to improve the penetration length accuracy of the forward projection. Then, the fitting methods and parameters suitable for the blades were derived and analyzed using the proposed framework, and the fitting model of the Hermite function was used to improve the fitting accuracy. Finally, the iterative fitting method based on the Hermite function model and polynomial fitting method were used to fit the blade data to obtain the optimal fitting curves. The obtained curves were used to correct the aero-engine turbine blade projections to reconstruct several sets of tomographic images. The results of the quantitative analysis of several groups of tomographic images with three image evaluation indicators show that the RMSE 0.0133 of the corrected tomographic images with the proposed method is lower than that of the compared methods, and PSNR 37.7050 dB and FSIM 0.9881 of the proposed method are higher than that of the compared methods. This proves that the iterative linear fitting correction technical framework can reduce the hardening artifacts of ICT images, improve image quality, and have strong robustness, which is consistent with visual observation. This work provides a new idea for imaging and detecting the cermet blades of new-generation aero engines.

## Figures and Tables

**Table 1:** Fitting MSE and time for different n values.

*n*	1	2	3	5	6	8	10
MSE	386.05	182.77	182.98	181.24	180.23	179.33	178.86
Time	1.39 s	2.31 s	2.97 s	3.98 s	4.22 s	4.99 s	5.52 s

**Table 2:** RMSE, PSNR, and FSIM of images in Figure 10a–g.

	Uncorrected	Polynomial 6	Polynomial 5	Polynomial 4	Polynomial 3	Akima 5	Hermite 5
RMSE	0.2665	0.1015	0.0641	0.0365	0.0227	0.0221	0.0133
PSNR	11.6906 dB	20.0799 dB	24.0727 dB	28.9714 dB	33.0970 dB	33.3205 dB	37.7050 dB
FSIM	0.8838	0.9801	0.9820	0.9836	0.9782	0.9814	0.9881

## Data Availability

The data presented in this study are available on request from the corresponding author.

## Appendix A. Image Quantitative Evaluation Indicators

RMSE is one of the indicators used to evaluate image quality [28]. The calculated value of RMSE represents the difference between the reference image and the reconstructed image. The smaller the RMSE value, the closer the reconstructed image is to the reference image. Equation (A1) is the calculation formula of RMSE.

(A1) $$RMSE=\sqrt{\frac{1}{M\times N}\overset{M}{\underset{i=1}{\sum }}\sum_{j=1}^{N}{\left(\rho^{\prime }\left(i,j\right)-\rho \left(i,j\right)\right)}^{2}}$$

where $\rho^{\prime }$ is the reference image, and ρ is the reconstructed image.

PSNR is one of the indicators to evaluate image quality [29]. It is based on the pixel error of the whole image to express the quality of the reconstructed image and the comparison of the reference image. As shown in Equation (A2), the larger the PSNR, the better the image quality.

(A2) $$PSNR=10lg[\frac{MAX^{2}}{MSE(\rho^{\prime },\rho )}]$$

where MAX is the maximum pixel gray value (for example, 255 for an 8-bit image), and MSE is the mean square error.

FSIM is one of the indicators to evaluate image quality [30]. FSIM summarizes three factors, including the brightness, contrast, and structure of the image, to evaluate the image quality by calculating the similarity of these factors. The larger the FSIM, the closer the reconstructed image is to the reference image. Equation (A3) is the FSIM calculation formula.

(A3) $$FSIM=\frac{\sum_{x\in \Omega }S_{L}(\rho )PC_{m}(\rho )}{\sum_{x\in \Omega }PC_{m}(\rho )}$$

where $S_{L}(x)$ is the local quality mapping in the reconstructed image ρ, and $PC_{m}(x)$ is the phase consistency of the reconstructed image ρ.
